# *O*-GlcNAc modification: why so intimately associated with phosphorylation?

**DOI:** 10.1186/1478-811X-9-1

**Published:** 2011-01-11

**Authors:** Suresh Mishra, Sudharsana R Ande, Neil W Salter

**Affiliations:** 1 Department of Internal Medicine University of Manitoba, Winnipeg, Canada; 2 Department of Physiology, University of Manitoba, Winnipeg, Canada

## Abstract

Post-translational modification of proteins at serine and threonine side chains by β-*N*-acetylglucosamine (*O*-GlcNAc) mediated by the enzyme β-*N*-acetylglucosamine transferase has been emerging as a fundamental regulatory mechanism encompassing a wide range of proteins involved in cell division, metabolism, transcription and cell signaling. Furthermore, an extensive interplay between *O*-GlcNAc modification and serine/threonine phosphorylation in a variety of proteins has been reported to exist. However, our understanding of the regulatory mechanisms involved in *O*-GlcNAc modification and its interplay with serine/threonine phosphorylation in proteins is still elusive. Recent success in the mapping of *O*-GlcNAc modification sites in proteins as a result of technological advancement in mass spectrometry have revealed two important clues which may be inherently connected to the regulation of *O*-GlcNAc modification and its interplay with phosphorylation in proteins. First, almost all *O*-GlcNAc modified proteins are known phospho proteins. Second, the prevalence of tyrosine phosphorylation among *O*-GlcNAc modified proteins is exceptionally higher (~68%) than its normal occurrence (~2%) alone. We hypothesize that phosphorylation may be a requisite for *O*-GlcNAc modification and tyrosine phosphorylation plays a role in the interplay between *O*-GlcNAc modification and serine/threonine phosphorylation in proteins. In other words, the interplay between *O*-GlcNAc modification and phosphorylation is not limited to serine/threonine phosphorylation but also includes tyrosine phosphorylation. Our hypothesis provides an opportunity to understand the underlying mechanism involved in *O*-GlcNAc modification and its interplay with serine/threonine phosphorylation in proteins. Furthermore, implication of our hypothesis extends to tyrosine kinase signaling.

## Background

*O*-GlcNAc cycling in proteins, mediated by the enzymes *O*-GlcNAc transferase (OGT) and *O*-GlcNAc amidase (OGA), is dynamically regulated in response to various stimuli and is remarkably similar to phosphorylation [1]. It has been more than 25 years since it was first discovered by Torres and Hart [2]. However, relative development in this field has remained sluggish for almost two decades, mainly due to the lack of tools and techniques for the identification and quantification of *O*-GlcNAc modification in proteins. As a result, our knowledge of the site-specific functions of *O*-GlcNAc modified proteins is very limited. Development of an *O*-GlcNAc specific antibody in 2001 by Hart and colleagues [3] has added some momentum in this field and contributed significantly in the identification of *O*-GlcNAc modified proteins. As *O*-GlcNAc modification in protein occurs at serine/threonine residues, the potential for interplay between serine/threonine phosphorylation and *O*-GlcNAc modification has been realized very early on [4]. Then it was indeed found to be the most common feature associated with *O*-GlcNAc modification and attributed to the further development in this area [5-7]. However, unlike phosphorylation which is regulated by hundreds of kinases and phosphatases, *O*-GlcNAc cycling has only two mediators: OGT and OGA [1]. This would imply that there must be fundamental differences in the way *O*-GlcNAc modification in proteins is regulated in relation to phosphorylation.

## Why the Occurrence of Tyrosine Phosphorylation among *O*-GlcNAc Modified Proteins is Exceptionally High

**Figure 1:**
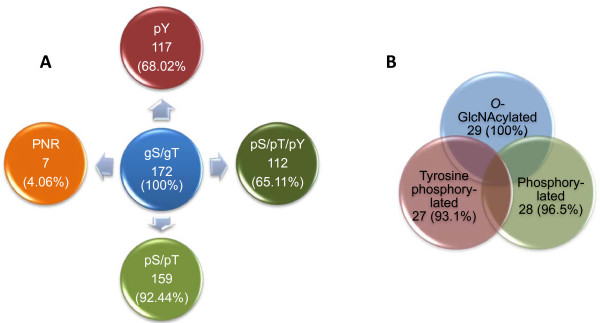
**Venn diagrams showing exceptionally high prevalence of tyrosine phosphorylation in *O*-GlcNAc modified proteins**. (A) Serine, threonine and tyrosine phosphorylation status of all known *O*-GlcNAc modified proteins curated at PhosphoSitePlus^® ^http://www.phosphosite.org (B) Phosphorylation (i.e. pS/pT/pY) and tyrosine phosphorylation status of dynamically altered *O*-GlcNAc modified (a total of 29 proteins) in response to inhibition of glycogen synthase kinase-3 (GSK-3). gS, GlcNAcylated serine; gT, GlcNAcylated threonine; pS, phosphorylated serine; pT, phosphorylated threonine; pY, phosphorylated tyrosine; PNR, phosphorylation not reported yet.

Earlier evidence regarding the interplay between *O*-GlcNAc modification and serine/threonine phosphorylation has pointed towards an inverse relationship between these two modifications [4-6]. However, emerging evidence suggests that the relationship between *O*-GlcNAc modification and serine/threonine phosphorylation is more extensive than initially thought [7,8]. Recently we have reported that tyrosine phosphorylation interacts with *O*-GlcNAc modification, a phenomenon which was previously not known [9]. Subsequently, two more articles were published showing that *O*-GlcNAc modification of insulin receptor substrate 1 (IRS1) occurs in close proximity of tyrosine phosphorylation sites and affects the tyrosine phosphorylation dependent function of IRS1 [10,11]. Taken together, these evidences would suggest that the interaction between *O*-GlcNAc modification and phosphorylation is not limited to serine/threonine phosphorylation (as initially thought) though rather also includes tyrosine phosphorylation. To further substantiate our hypothesis of the interaction between tyrosine phosphorylation and *O*-GlcNAc modification, we analyzed the tyrosine phosphorylation status of all *O*-GlcNAc modified proteins curated at PhosphoSitePlus^® ^http://www.phosphosite.org along with phosphoproteomes [12]. Analysis of *O*-GlcNAc modified proteins revealed that 68.02% of them are known to be tyrosine phosphorylated (Figure 1A). As our knowledge of phosphoproteomes is currently increasing rapidly it is expected that this percentage will increase further. Most importantly, 65.11% of the *O*-GlcNAc modified proteins were found to be serine/threonine and tyrosine phosphorylated (Figure 1A). This would mean that all *O*-GlcNAc modified proteins which are tyrosine phosphorylated are also serine/threonine phosphorylated. Further analysis of motifs around *O*-GlcNAc modification sites revealed that in the majority of the cases these dynamic modifications occur in close proximity of each other. It is of note that the common features surrounding *O*-GlcNAc modification sites described here and before [9], which are apparent in the primary structure of a protein, can also be achieved or constituted in the secondary structure of proteins by residues present distantly from each other. Intriguingly such a high prevalence of tyrosine phosphorylation among *O*-GlcNAc modified proteins, which is many folds higher than its normal occurrence [~2%, [13]], further supports our hypothesis of a role of tyrosine phosphorylation in this dynamic process. Furthermore, a recent report on the recruitment of OGT in response to insulin stimulation and the subsequent *O*-GlcNAc modification of insulin signaling intermediates as a part of an intrinsic mechanism involved in the attenuation of insulin's tyrosine phosphorylation dependent signaling also support our hypothesis [14].

## Is Phosphorylation a Requisite for *O*-GlcNAc Modification

The wide range of simultaneous changes in *O*-GlcNAc modification in a number of proteins under different experimental conditions as previously observed by Wang *et al *[15] and in a recent report by Gu *et al *[16], could not be explained by changes in the expression and/or the activity of *O*-GlcNAc cycling enzymes. Especially when there is only one OGT and one OGA [1]. It is possible that the phosphorylation status of proteins is the major determinant of subsequent *O*-GlcNAc modification (i.e. regulation directed by the substrate itself). This may be mediated by the recruitment of various interacting partners in the form of a protein complex, which may modulate the binding and/or the catalytic activity of *O*-GlcNAc cycling enzymes. A prerequisite for this hypothesis is that all *O*-GlcNAc modified proteins must be phosphoproteins. To confirm if this is the case, we checked the phosphorylation status of all *O*-GlcNAc modified proteins curated at PhopshoSitePlus^® ^database along with phosphoproteomes [12]. Interestingly, ~93% of the *O*-GlcNAc modified proteins are known phosphoproteins (Figure 1A). The phosphorylation status of ~4% of the *O*-GlcNAc modified proteins is not known (Figure 1A). As the number of proteins in phosphoproteomes are currently increasing rapidly it is expected that this minor gap will be further dwindled down. Therefore, a possibility of phosphorylation as a requisite for *O*-GlcNAc modification may not be ruled out.

It appears that *O*-GlcNAc modification of proteins occurs in a small subset of phosphoproteins. This raises another question: what makes a small subset of phosphoproteins able to undergo *O*-GlcNAc modification? As tyrosine phosphorylation has been shown to facilitate *O*-GlcNAc modification [9], it is possible that the presence of a phosphorylated tyrosine residue may significantly enhance the possibility of subsequent *O*-GlcNAc modification in a protein. Furthermore, it is possible that the *O*-GlcNAc modification status of a protein at any given time may be regulated by the overall phosphorylation status of that particular protein. If this is true then the perturbation of the phosphorylation status in proteins should drastically alter *O*-GlcNAc modification. To substantiate our hypothesis we analyzed a recent study by Wang *et al *[15] on a systemic glycoproteomics analysis in response to inhibition of glycogen synthase kinase-3 (GSK-3), an important kinase involved in many signaling pathways. In this study a total of 45 *O*-GlcNAc modified proteins were identified [15]. By quantitative measurements the authors confirmed that at least 10 proteins had an apparent increase of *O*-GlcNAc modification, whereas 19 others proteins showed decreases [15]. To get an insight into the dynamic relationship between tyrosine phosphorylation and *O*-GlcNAc modification, we examined the tyrosine phosphorylation status all *O*-GlcNAc modified proteins (at http://www.phosphosite.org) that have been identified to undergo significant changes in *O*-GlcNAc modification in response to GSK-3 inhibition. As expected, 28 (96.5%) out of 29 proteins are known phosphoproteins (Figure 1B). Most importantly, 27 (93.1%) out of 29 proteins are also known tyrosine phosphorylated proteins (Figure 1B). Such a high prevalence of *O*-GlcNAc modification and tyrosine phosphorylation together strongly support our hypothesis of a dynamic relationship between tyrosine phosphorylation and *O*-GlcNAc modification and a potential role of tyrosine phosphorylation in the interaction between *O*-GlcNAc modification and serine/threonine phosphorylation which warrants further investigation.

## Implications

Our hypotheses provide an opportunity to understand the regulatory mechanisms involved in *O*-GlcNAc modification of proteins and will be helpful in answering some of the unanswered questions in this field such as: i) Why *O*-GlcNAc modification in proteins is so intimately associated with phosphorylation? ii) Why prevalence of tyrosine phosphorylation among *O*-GlcNAc modified proteins are many fold higher than its normal occurrence? iii) Is phosphorylation a requisite for *O*-GlcNAc modification? iv) Does *O*-GlcNAc modification work in a concerted manner with phosphorylation or does it have an exclusive function? Implication of our hypothesis also extends to tyrosine kinase signaling such as growth factors and immune receptor signaling and may lead to the development of a new paradigm in tyrosine kinase signaling. It is anticipated that these hypotheses will stimulate research in this under-studied area and advance our understanding of the regulation and function of *O*-GlcNAc modified proteins.

## Competing interests

The authors declare that they have no competing interests.

## Authors' contributions

SM wrote the manuscript and contributed in data interpretation, SRA and NWS contributed in writing and data acquisition. All authors have read and approved the manuscript.
